# Variation of UVB, UVA, and blue-violet visible light transmittance in sun-protective shirts from the Brazilian market, after 50 washing cycles^؆^

**DOI:** 10.1016/j.abd.2026.501406

**Published:** 2026-06-20

**Authors:** Ana Cláudia Cavalcante Espósito, Daniel Pinho Cassiano, Felipe Kesrouani Lemos, Luana Diamante Domingues, Cristhiana Kise Saito, Hélio Amante Miot

**Affiliations:** a Department of Infectology, Dermatology, Imaging Diagnosis, and Radiotherapy, Faculty of Medicine, Universidade Estadual Paulista, Botucatu, SP, Brazil; b Hospital Regional de Presidente Prudente, Presidente Prudente, SP, Brazil; c Department of Dermatology, Escola Paulista de Medicina, Universidade Federal de São Paulo, São Paulo, SP, Brazil; d Faculdade de Ciências e Tecnologia, Universidade Estadual Paulista, Presidente Prudente, SP, Brazil

Dear Editor,

Although widespread use, the effectiveness of topical sunscreen is limited by poor adherence and under-application, especially during intense exposure, such as sports and occupational activities.^1^, ^2^ Photoprotection by clothing relies on fabrics blocking or attenuating Ultraviolet Radiation (UVR) before it reaches the skin, providing a continuous barrier without the need for reapplication.^3^

The UV-blocking capacity of a fabric is graded by its Ultraviolet Protection Factor (UPF).^2^, ^3^, ^4^ Garments classified as providing excellent photoprotection are generally defined as UPF50 or higher, corresponding to ≤ 2% UVB transmittance.^3^, ^4^, ^5^ However, for protection against UVA radiation and visible light, there are still no regulatory standards for determining specific protection factors.

In the United States, certification simulates about two years of garment use, including testing the transmittance after 40 washes and exposure to sunlight and chlorinated water.^4^ In Brazil, however, there is no mandatory certification, and the durability of UV-protective clothing under real-use conditions remains unknown. Therefore, this study evaluated changes in UVB, UVA, and blue-violet light transmittance after 50 washing and sun-drying cycles in Brazilian UV-protective shirts.

Six commercially available long-sleeve black shirts, manufactured with fabrics (UPF50+) indicated for UV protection (Table 1), were submitted to 50 household machine wash cycles (cold water, detergent, and fabric softener), followed by direct sun-drying for at least 4-hours. Shirt fabric samples were photographed under light microscopy before washing (T0), and after 10 (T10), 30 (T30), and 50 (T50) wash cycles. UVB, UVA, and blue-violet visible light (400–500 nm range). Using direct sunlight at noon under clear-sky conditions, transmittance was evaluated at baseline (T0) and after 10 (T10), 30 (T30), and 50 (T50) washing cycles. All measurements were performed outdoors in the same open area to ensure consistent environmental exposure. The sensors were positioned perpendicularly to the incident solar radiation and stabilized on a fixed support to avoid angular variation. For each measurement, incident irradiance was first recorded without the fabric. Subsequently, the fabric sample was placed flat and taut over the sensor aperture, avoiding folds or shadowing, and transmitted irradiance was measured immediately thereafter to minimize fluctuation in solar intensity. UVB transmittance was assessed using a UVB Digital Ultraviolet Radiometer (ZooMed – San Luis Obispo, CA, USA), UVA transmittance using a Digital Ultraviolet Radiometer 4.2 UVA (Solarmeter – Glenside, PA, USA), and blue-violet visible light (400–500 nm) transmittance using an RD-7 Radiometer (Ecel – Ribeirão Preto, SP, Brazil), according to the manufacturers’ specifications. Three consecutive measurements were obtained for each shirt at each time point, and the mean value was used for statistical analysis.^5^, ^6^, ^7^, ^8^, ^9^

**Table 1:** Description of the textile composition of each of the six brands of photoprotective shirts analyzed.

Sample	Textile composition	Brand
Shirt 1	100% polyamide	Speedo
Shirt 2	90% polyamide + 10% elastane	Fila
Shirt 3	80% polyamide + 20% elastane	Puma
Shirt 4	100% polyester	Columbia
Shirt 5	91% polyamide + 9% elastane	UV LINE
Shirt 6	100% polyester	Nike

Fig. 1 presents microscopic photographs of the interweave structures of the six brands studied before washing (T0) and after 50 cycles (T50), and Fig. 2 demonstrates the evolution of ultraviolet transmittance, fabric opening area, and the estimated UPF of the six photoprotective shirts across washing cycles (T0, T10, T30, and T50).

**Fig. 1:**
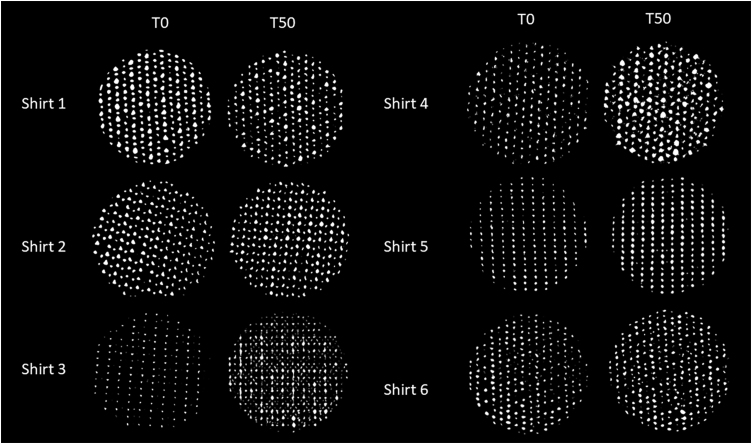
Microscopic photograph (40× magnification) of the interweave structures of the six studied brands before washing (T0) and after 50 washing cycles (T50). Following repeated washing and sun-drying cycles, structural changes are observed in several samples, including modifications in fiber organization and inter-fiber spacing. The heterogeneity of these alterations across brands may partially explain the variability detected in ultraviolet transmittance and estimated UPF, reinforcing the influence of textile composition and structural stability on photoprotective performance.

**Fig. 2:**
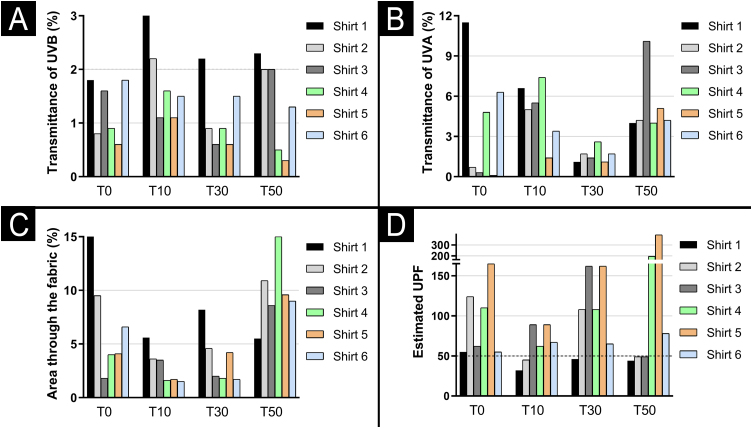
Measurements of the fabric's optical and structural parameters after repeated washing cycles. (A) UVB transmittance (%); (B) UVA transmittance (%); (C) Percentage of open area through the fabric; and (D) Estimated ultraviolet protection factor (estimated UPF = 1/Transmitance) of six UV-protective black long-sleeve shirts at baseline (T0) and after 10 (T10), 30 (T30), and 50 (T50) washing cycles. Dashed line: UPF 50.

UVB transmittance remained globally low (≤ 2%) in all but shirt 1, although variability was observed between brands and throughout the washing cycles. UVA transmittance showed high variability between brands and over time. Shirts 1 and 6 showed an initial reduction in transmittance after 10 washes, possibly due to fabric compaction, while shirt 3 showed a major increase after 50 cycles, suggesting structural degradation or changes in the material's optical properties. Blue-violet light transmittance was negligible for all shirts at all time points.

Fabric opening area demonstrated a biphasic pattern, with reduction after 10 washes (T10) in all samples, except for shirt 3, suggesting compaction or fabric shrinkage, followed by increases at T50 except for shirt 1.

Estimated UPF behavior was heterogeneous across samples. Some shirts, such as 4 and 5, showed an increase in UPF after multiple washings, possibly related to initial fabric post-washing modification, whereas others demonstrated a reduction after 50 cycles. Shirt 1 (polyamide) performed below UPF50 at T10, T30, and T50. These findings reinforce that photoprotective performance does not depend solely on the number of washes, but also on textile composition and the structural response of each material to mechanical wear.

Clothing not specifically labeled as photoprotective may also block UVR with varying efficacy, and the amount of dye incorporated into the fabric appears to play a more important role in photoprotection than color alone. Additionally, polyester fabrics, including polyester-cotton blends, frequently exhibit UPF values above 50 due to their low porosity.^3^, ^4^, ^5^, ^10^

Currently, photoprotective garments are designed based on textile composition, fabric density, and dyeing patterns rather than relying on chemical UV-blocking additives. They are predominantly made from synthetic fibers such as polyester and nanofibers, woven to form highly compact structures that significantly reduce the “hole effect”. Moreover, UV-protective clothing often employs highly saturated textile dyes.^4^

Synthetic fibers tend to offer greater intrinsic UV protection compared with natural fibers. For example, polyester and polyamide fabrics generally block more UV radiation than cotton of the same thickness and density. Polyester contains aromatic rings in its molecular structure, which absorb UV radiation. In contrast, cotton and other natural fibers, especially when white, lightweight, or bleached, are less effective.^2^, ^5^, ^10^, ^11^ Elastic fabrics may also show reduced UPF, since stretching increases UV penetration.^3^

In a similar study from the USA, the authors washed seven garments up to 50 cycles and measured changes in UPF. While most maintained stable values through repeated washing, two brands showed a notable decrease in UPF, including one that used a nano-zinc UV varnish. In contrast, garments that did not rely on added chemical finishes tended to sustain their UPF more consistently, with one maintaining the maximum measurable UPF even after 50 washes. These results suggest that some UV-protective varnishes may deplete or wash out over time, raising questions about their long-term necessity and whether textile construction alone might offer more durable photoprotection.^11^

In a study that compared *in vivo* sun-protective fabrics with commercial sunscreens, all tested textiles provided superior and more consistent UV protection, blocking more UVA and UVB radiation than SPF 30 and 50 sunscreens. The findings support prioritizing photoprotective clothing as a primary defense against UV exposure, with sunscreen serving as an important complementary measure.^12^

The limitations include: a small sample size, limited to the Brazilian market, the use of natural sunlight rather than spectrophotometry, and the exclusive evaluation of dry black shirts. Shirts were not evaluated under conditions involving sweating, stretching, or fabric tearing, which occur during field use.^13^, ^14^

Concluding, the evaluated UV-protective shirts generally preserved low sun-radiance transmittance after 50 washing cycles; however, performance was heterogeneous, and one product failed to maintain UPF ≥50 throughout follow-up. The observed variability reinforces the need for nationally standardized certification protocols that include durability testing to ensure consistent and reliable photoprotection for consumers, particularly those engaged in outdoor activities and/or at high risk for skin cancer.

## Authors’ contributions

Ana Cláudia Cavalcante Espósito: Study conception and design; data analysis and interpretation; drafting of the manuscript; critical literature review; critical revision of the manuscript; approval of the final version of the manuscript.

Daniel Pinho Cassiano: Data analysis and interpretation; drafting of the manuscript; data acquisition; approval of the final version of the manuscript.

Felipe Kesrouani Lemos: Data analysis and interpretation; statistical analysis; drafting of the manuscript; data acquisition; approval of the final version of the manuscript.

Luana Diamante Domingues: Data analysis and interpretation; drafting of the manuscript; data acquisition; approval of the final version of the manuscript.

Cristiana Kise Saito: Data analysis and interpretation; drafting of the manuscript; data acquisition; approval of the final version of the manuscript.

Hélio Amante Miot: Study conception and design; data analysis and interpretation; statistical analysis; drafting of the manuscript; critical literature review; critical revision of the manuscript; approval of the final version of the manuscript.

## Declaration of Generative AI and AI-assisted technologies in the writing process

During the preparation of this work, the authors used ChatGPT 5.0 to assist with English language editing. After using this tool, the authors reviewed and edited the content as needed and take full responsibility for the content of the published article.

## Financial support

10.13039/501100003593CNPq (306358/2022-0) – Hélio Amante Miot is a CNPq researcher.

## Research data availability

Does not apply.

## Conflicts of interest

None declared.

## References

[1] Buller D.B., Andersen P.A., Walkosz B.J., Scott M.D., Maloy J.A., Dignan M.B. (2012). Compliance with sunscreen advice in a survey of adults engaged in outdoor winter recreation at high-elevation ski areas. J Am Acad Dermatol.

[2] Schalka S., Steiner D., Ravelli F.N., Steiner T., Terena A.C., Marçon C.R. (2014). Brazilian consensus on photoprotection. An Bras Dermatol.

[3] Boothby-Shoemaker W.T., Mohammad T.F., Ozog D.M., Lim H.W. (2022). Photoprotection by clothing: A review. Photodermatol Photoimmunol Photomed.

[4] Lu J.T., Ilyas E. (2022). An overview of ultraviolet-protective clothing. Cureus.

[5] Yoshida M.M., Esposito A.C.C., Miot H.A. (2020). UVB, UVA, and visible light (blue-violet range) transmittance of clothing used in Brazil. An Bras Dermatol.

[6] Mazeto I.F.D.S., Esposito A.C.C., Cassiano D.P., Miot H.A. (2022). Sun exposure (UVB, UVA, and blue-violet visible light) in ordinary daily situations. Int J Dermatol.

[7] Heintze S.D., Cavalleri A., Peschke A., Schüpbach P. (2005). Fluorescence microscopy for the evaluation of the margins of Class V restorations in vitro. J Adhes Dent.

[8] Diehl J.J.E., Baines F.M., Heijboer A.C., van Leeuwen J.P., Kik M., Hendriks W.H. (2018). A comparison of UVb compact lamps in enabling cutaneous vitamin D synthesis in growing bearded dragons. J Anim Physiol Anim Nutr (Berl).

[9] Cusack L., Rivera S., Lock B., Benboe D., Brothers D., Divers S. (2017). Effects of a light-emitting diode on the production of cholecalciferol and associated blood parameters in the bearded dragon (*Pogona vitticeps*). J Zoo Wildl Med.

[10] Saha B., Saha A., Das P., Kakati A., Banerjee A., Chattopadhyay P. (2024). A comprehensive review of ultraviolet radiation and functionally modified textile fabric with special emphasis on UV protection. Heliyon.

[11] Fernau E., Ilyas S.M., Ilyas E.N. (2023). The impact of routine laundering on Ultraviolet Protection Factor (UPF) values for commercially available sun-protective clothing. Cureus.

[12] Berry E.G., Bezecny J., Acton M., Sulmonetti T.P., Anderson D.M., Beckham H.W. (2022). Slip versus sop: A head-to-head comparison of UV-protective clothing to sunscreen. Cancers (Basel).

[13] Aguilera J., Navarrete-de Gálvez E., Sánchez-Roldán C., Herrera-Ceballos E., de Gálvez M.V. (2023). Sun-protective properties of technical sportswear fabrics 100% polyester: the influence of moisture and sweat on protection against different biological effects of Ultraviolet (UV) radiation. Photochem Photobiol.

[14] Gambichler T., Hatch K.L., Avermaete A., Bader A., Herde M., Altmeyer P. (2002). Ultraviolet protection factor of fabrics: comparison of laboratory and field-based measurements. Photodermatol Photoimmunol Photomed.

